# Common Variable Immunodeficiency and Gastric Malignancies

**DOI:** 10.3390/ijms19020451

**Published:** 2018-02-02

**Authors:** Patrizia Leone, Angelo Vacca, Franco Dammacco, Vito Racanelli

**Affiliations:** Department of Biomedical Sciences and Human Oncology, Unit of Internal Medicine, University of Bari Medical School, 70124 Bari, Italy; patrizia.leone@uniba.it (P.L.); angelo.vacca@uniba.it (A.V.); francesco.dammacco@uniba.it (F.D.)

**Keywords:** common variable immunodeficiency, gastric cancer, human immunoglobulins, lymphoproliferative disorders

## Abstract

Common variable immunodeficiency (CVID) is an immunodeficiency disorder with a high incidence of gastrointestinal manifestations and an increased risk of gastric carcinoma and lymphoma. This review discusses the latest advancements into the immunological, clinical and diagnostic aspects of gastric malignancies in patients with CVID. The exact molecular pathways underlying the relationships between CVID and gastric malignancies remain poorly understood. These include genetics, immune dysregulation and chronic infections by *Helicobacter pylori*. Further studies are needed to better stratify the risk for cancer in these patients, to elaborate surveillance programs aimed at preventing these complications, and to develop new and more effective therapeutic approaches.

## 1. Introduction

Common variable immunodeficiency (CVID) comprises a heterogeneous group of relatively rare disorders characterized by remarkable decrease of two or three major immunoglobulin isotypes (IgG, IgA, and IgM), often associated to defects in cell-mediated immunity [1]. Several immunological studies of large cohorts of CVID patients have demonstrated phenotypic and functional abnormalities of B cells [2,3], T cells [4,5,6], and antigen-presenting cells [7,8]. These abnormalities include mutations that occur in genes essential for the co-operation between B and T cells in the germinal center, as well as for intrinsic signaling pathways of such cells. A flow cytometry-based analysis of CVID patients revealed a marked reduction of mature class-switched CD27^+^IgD^−^IgM^−^ memory B cells and/or an increased number of CD19^+^CD21^−^ immature B cells [3]. In addition, a decreased number of CD4^+^ naïve T cells and regulatory T cells has been detected in the patients’ peripheral blood, possibly ascribable to defective generation of T cell precursors in the bone marrow [4]. Bone marrow biopsies have in fact shown the absence or a significant decrease of plasma cells and, conversely, an increase of diffuse and nodular T cell infiltrates [9].

The diagnosis of CVID obviously requires the exclusion of other causes of hypogammaglobulinemia [10]. The mean age at diagnosis is 29 years for males and 33 years for females, although the condition can be diagnosed at any age without gender predominance [11]. The prevalence varies widely worldwide, ranging from 1:100,000 to 1:10,000 of the general population [12]. Although CVID is clinically highly variable and heterogeneous, severe, recurrent, and chronic bacterial infections of the respiratory and gastrointestinal tracts are the major characterizing features. In addition, approximately half of the patients suffer from non-infectious complications, including autoimmune, lung and gastrointestinal disease, benign lymphoproliferation, and malignancies [11]. In particular, the gastrointestinal tract [13] and the lymphoid tissue are among the most affected systems [14,15,16], as shown by the fact that CVID patients have an almost 47-fold increased risk for gastric cancer and a 30-fold increased risk for lymphoma [17]. 

In this paper, we will provide an overview of the main clinical, diagnostic and immunological features of gastric malignancies in patients with CVID, with special emphasis for gastric carcinoma and lymphoproliferative disorders.

## 2. Genetic Abnormalities

CVID is a polygenic disease which is the result of numerous genetic immune defects, summarized in Table 1. The mode of inheritance is mostly autosomal dominant, and autosomal recessive in about 20% of the cases. The most frequently identified mutations have been discovered in the tumor necrosis factor (TNF) receptor superfamily member 13B (TNFRSF13B) gene encoding TACI (transmembrane activator and calcium-modulating cyclophilin ligand interactor), a B cell-specific TNF receptor superfamily member. Both homozygous and heterozygous coding variants have been identified in patients with CVID [18,19]. TACI is preferentially expressed on marginal zone B cells, CD27^+^ memory B cells, and plasma cells. Its ligands are the B cell-activating factor (BAFF) and the proliferation-inducing ligand (APRIL) involved in cell survival, apoptosis, and isotype switching [20]. 

Defects of TACI impair BAFF and APRIL signaling, and consequently plasma cell survival, maturation and class switch recombination, Ig production [21,22], and the removal of autoreactive B cells at the central B cell tolerance checkpoint, thus increasing the susceptibility of CVID patients to autoimmune diseases [23]. A181E and C104R are the two most frequent TNFRSF13B variants in these patients [18,19,24], although the mentioned mutations are neither necessary nor sufficient to cause CVID. While healthy individuals with a single pathogenic TNFRSF13B allele have a normal B cell phenotype, this is not the case for CVID patients with the same TNFRSF13B status, suggesting that the disease expression depends on the loss of compensating forces. Further studies are needed to elucidate the additional genetic and environmental factors that act in concert to generate the disease.

Homozygous and heterozygous mutations in the BAFF receptor (BAFF-R) gene, and single nucleotide polymorphisms resulting in BAFF-R missense mutations have been reported in CVID patients [25]. BAFF-R is required for B cell maturation and survival [26], and its mutations are associated to impairment in the proliferation, differentiation, and maturation of B lymphocytes [27]. Animal studies, carried out with both knockout and transgenic models, demonstrated that disruption of BAFF-R results in an immunological phenotype similar to that observed in CVID, suggesting that BAFF-R may be involved in the pathogenesis of CVID [28]. 

Biallelic deleterious mutations in members of the CD19 B-cell receptor complex (CD19, CD21, and CD81) and CD20 could also be involved in the onset of CVID [29,30,31]. Homozygous deletion in the inducible co-stimulator (ICOS) gene has been associated with adult-onset CVID [32]. ICOS belongs to the family of co-stimulatory T cell molecules and is expressed by antigen-activated T cells. Its unique ligand is ICOS-L expressed constitutively on B cells [33]. ICOS:ICOS-L interaction plays an important role in mediating T-B cell cooperation and promoting the terminal differentiation of B cells into memory cells and plasma cells. Patients with ICOS deletion displayed a reduced number of naïve, switched and memory B cells as well as low serum Ig levels [32]. This is further supported by the results achieved in ICOS and ICOS-L knockout mice, both showing a defect in germinal center formation and in humoral immune responses, due to the lack of T cell-mediated help to the B cells [34].

TNFRSF13B, tumor necrosis factor receptor superfamily member 13B; BAFF-R, B cell-activating factor receptor; ICOS, inducible costimulatory; CARD, caspase activation and recruitment domain; Bob1, B cell-specific transcriptional co-activator; ADAM, disintegrin and metalloproteinas genes; CTLA4, cytotoxic T lymphocyte antigen-4; PIK3CD, phosphatidylinositol-4,5-bisphosphate 3-kinase catalytic subunit delta; NFκB2, nuclear factor kappa B2; PLCG2, phospholipase C gamma 2; LRBA, lipopolysaccharide-responsive beige-like anchor.

The described mutations, however, account for less than 15% of CVID cases. The remaining 85% of the patients do not have a known genetic defect and it is likely that other genes besides those already identified may be involved in the pathogenesis of the CVID. For example, single nucleotide polymorphisms in genes implicated in deoxyribonucleic acid (DNA) repair (MSH5, MSH2, MLH1, RAD50, and NBS1) and in genes involved in B cell development, encoding the caspase activation and recruitment domain (CARD)11 and the B cell-specific transcriptional co-activator Bob1, could be associated with CVID [35,36]. Furthermore, a genome-wide association study, using single nucleotide polymorphism arrays and copy number variation, revealed a strong relationship between CVID and the MHC region as well as between CVID and a disintegrin and metalloproteinase genes (ADAM) [37]. 

In addition, a CVID-like syndrome, characterized by hypogammaglobulinemia, progressive loss of circulating B cells, immune dysregulation, and lymphocytic infiltration of target organs was demonstrated to be caused by heterozygous mutations in: (a) cytotoxic T lymphocyte antigen-4 (CTLA4) involved in T and B lymphocyte homeostasis [38]; (b) phosphatidylinositol-4,5-bisphosphate 3-kinase catalytic subunit delta (PIK3CD) gene, resulting in hyperactivation of the PI3K signaling pathway important for B and T cell development, differentiation, and function [39]; (c) nuclear factor kappa B2 (NFκB2) required for B cell development and antibody production [40]; (d) phospholipase C gamma 2 (PLCG2) causing gain of PLCγ(2) function, a signaling molecule expressed in B cells [41]; (e) lipopolysaccharide-responsive beige-like anchor (LRBA) causing severe defects in B cell development and activation [42]; and (f) CD27, a lymphocyte costimulatory molecule implicated in B cell and plasma cell function, survival, and differentiation [43].

## 3. Common Variable Immunodeficiency and Gastric Cancer

Many studies reported an increased risk of gastric cancer in CVID patients [15,16,17,44,45]. The first evidence was described in 1985 when a prospective study of 220 patients with CVID followed for 11 years showed a 47-fold increased risk of stomach cancer [17]. A multicenter study which examined 176 Danish and Swedish patients with CVID and their relatives reported a risk of stomach cancer 10-fold higher for CVID subjects, whereas no increased risk was found for this or any other type of cancer among 626 relatives of CVID patients. This suggests that the increased risk of gastric cancer is related to the immunodeficiency per se, rather than to specific genetic alterations shared with their relatives [45]. In an analysis of the Australasian Society of Clinical Immunology and Allergy primary immunodeficiency disease registry of 1132 subjects from 79 centers, it was shown that only subjects with CVID and ataxia telangiectasia had an increased risk of cancer. A high relative risk in relation to an age-matched general population was observed for non-Hodgkin’s lymphoma (NHL), leukemia, and gastric cancer [16]. 

In a recent study, the incidence of cancer in patients with primary immunodeficiency diseases enrolled in the United States Immune Deficiency Network registry was assessed compared with age-adjusted cancer incidence in the Surveillance, Epidemiology and End Results Program database. An increased risk of NHL, gastric cancer, and skin cancer was observed in 1285 patients with CVID. Gastric cancer, in particular, was more common than expected (*n* = 2 in men and *n* = 3 in women vs. expected rates of *n* = 0.4 in men (*p* = 0.011) and *n* = 0.7 in women (*p* = 0.005)) [44].

**Figure 1:**
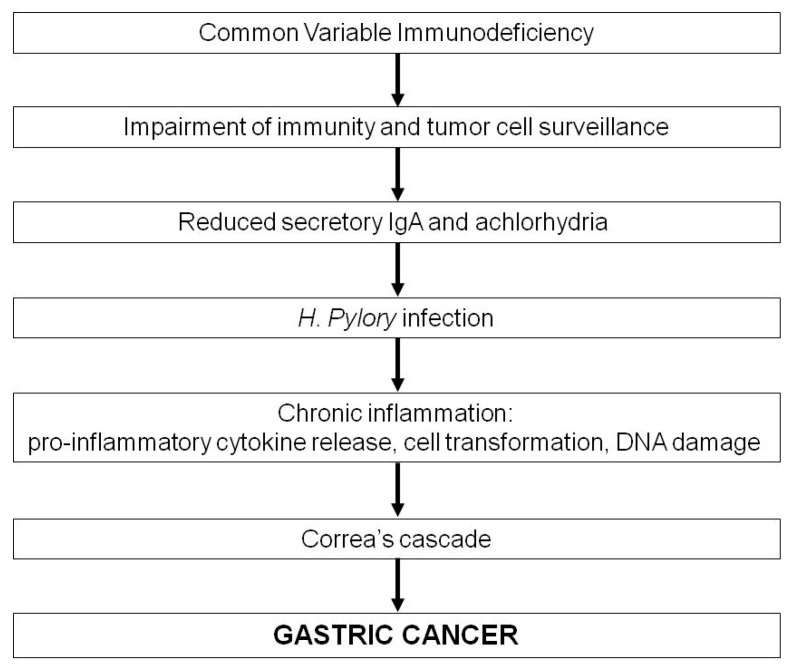
Hypothetical mechanisms of gastric cancer in patients with CVID.

The exact mechanisms underlying an enhanced frequency of gastric cancer in patients with CVID are unknown, but a possible sequence of events is schematized in Figure 1. The weakened immunity to potentially carcinogenic pathogens, such as *Helicobacter pylori* (HP), and the impaired tumor cell surveillance should obviously be considered predisposing factors to gastric cancer. Several studies have in fact ascribed the higher incidence of gastric cancer to HP infection (reviewed in [46]) and achlorhydria [17]. Eradication of HP in patients with non-atrophic gastritis has been demonstrated to prevent the subsequent development of gastric cancer [47]. Data from prospective studies revealed a two to nine fold increased risk of gastric cancer in the general population with HP infection [48,49]. More recently, however, gastric cancer appears rarer in CVID, possibly due to the more common use of antibiotics that would eradicate HP. Probably, the gastrointestinal defects associated with CVID, such as the decreased production of gastric IgA (with bactericidal activity against HP) and hydrochloric acid, may result in enhanced HP colonization and gastric inflammation, thus promoting carcinogenesis [50]. 

HP causes chronic gastritis by stimulating the release of pro-inflammatory cytokines and favoring achlorhydria; in addition, certain strains produce virulence factors with oncogenic effects on the gastric epithelium. It induces upregulation of oncogenes and silencing of tumor suppressor genes, triggering a stepwise cascade of events ranging from intestinal metaplasia to dysplasia to neoplasia, the so called Correa’s cascade (Figure 1) [51]. Moreover, an inflammatory response is generated, in that replicating cells are invaded by neutrophils and monocytes, which release reactive oxygen species (ROS) and reactive nitrogen species (RNS), thus inducing DNA breaks and point mutations in genes critical for cell replication and death [52]. It should also be emphasized that hypochlorhydria and achlorhydria may result in impaired defense against HP infection and enhanced bacterial overgrowth, including nitrate reducing strains, with consequent increased levels of N-nitroso compounds in the gastric juice. This endogenous nitrosation can promote the progression from gastric atrophy to intestinal metaplasia, dysplasia, and eventually carcinoma [46,53,54].

## 4. Features of CVID-Associated Gastric Cancer

CVID-associated gastric cancer typically displays peculiar features. First, it is diagnosed in patients younger than the overall gastric cancer population. Second, it is moderately to poorly differentiated intestinal-type adenocarcinoma, containing a high number of intra-tumoral lymphocytes. Third, it arises in a background of gastritis characterized by severe atrophy, pan-gastric distribution, intestinal metaplasia, plasma cell paucity, lymphoid nodular aggregates, and apoptotic activity [55]. This latter feature, which is reminiscent of gastritis from HP, may also underlie autoimmune gastritis given that autoimmunity is a well-known complication of CVID and pernicious anemia affects approximately 10% of patients [46]. Pernicious anemia is readily suspected by a low serum vitamin B12 and macrocytic red blood cells, although a precise diagnosis in CVID patients is more difficult because of the lack of typical anti-parietal cell and anti-intrinsic factor autoantibodies. Tissue damage in autoimmune gastritis is indeed mediated not only by autoantibodies targeting the parietal cell proton pump and intrinsic factor, but also by sensitized T cells. When fully developed, autoimmune gastritis displays dense and diffuse lymphoplasmacytic inflammation with the oxyntic epithelia replaced by atrophic (and metaplastic) mucosa, creating the phenotypic background in which gastric intestinal-type adenocarcinomas may arise [56,57]. Several studies have also addressed the role of HP infection in the pathogenesis of autoimmune gastritis, and there is evidence to support a mechanism of molecular mimicry between HP antigens and the proton pump [58]. Epidemiological studies suggest that a significant number of patients with autoimmune gastritis suffered from, or still have, HP infection and anti-proton pump autoantibodies have consistently been demonstrated in HP-infected patients.

An additional, potential abnormality that can be identified in patients with CVID-associated gastric cancer is granuloma resembling sarcoidosis [59]. Whether the granuloma reflects a primary T-cell defect, or an abnormal response to infectious agents is presently unknown. In any case, fungal and mycobacterial special stains are always appropriate when granulomas are identified, given that tuberculosis has been described in the setting of CVID [60].

## 5. Gastric Cancer Screening and Prevention in CVID Patients

Gastric cancer is the fourth most common cancer and the second leading cause of cancer death worldwide [61]. There is no ideal protocol for gastric cancer screening in high-risk CVID individuals, and prerequisites of screening programs differ from country to country because of the variable cancer incidence and mortality in each country, ethnic differences, and socio-economic conditions [62]. Consensus exists, however, over the usefulness of a risk assessment primarily based on the diagnosis of HP infection and/or pernicious anemia. 

Rather than on HP antibody test, the diagnosis of HP infection is usually based on urea breath test (UBT), stool antigen immunoassay and/or endoscopic biopsy. UBT is largely preferred being widely available, accurate, and noninvasive, with a sensitivity and specificity of roughly 90% [63]. An additional noninvasive method is the stool test, characterized by sensitivity in the range of 69–92% and specificity of approximately 75–89% [64]. HP infection is often asymptomatic, thus accounting for its uncommon detection and eradication at an early stage. Following its eradication, HP rarely recurs in the general population [65]. Whether this rare recurrence is also common to patients with CVID, given their lack of secretory IgA on the gastric mucosa, has not been established.

The diagnosis of pernicious anemia can be made by showing the presence of megaloblastic or macrocytic anemia, and measuring serum vitamin B_12_ and iron levels. Therefore, a screening protocol to target patients with CVID who are at the highest risk of gastric cancer should include three easy, non-invasive tests such as UBT, serum B_12,_ and serum iron [65]. 

**Figure 2:**
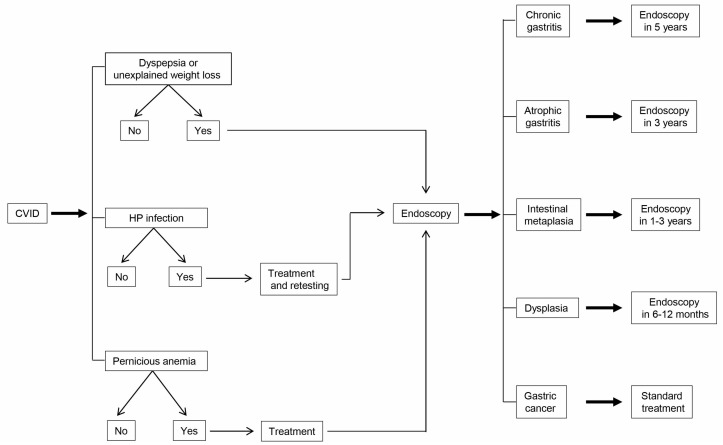
Protocol of screening and surveillance for gastric cancer in patients with CVID.

Regardless of the presence of pernicious anemia or HP infection, patients with CVID should be considered at increased risk for gastric cancer [15,16,17,44,45]. We propose a step-by-step evaluation of all patients with CVID, with invasiveness increasing stepwise according to the risk (Figure 2). The initial screening should focus on noninvasive tests, such as UBT to detect HP infection and the measurement of serum vitamin B_12_ and iron to detect the presence of pernicious anemia. If patients are positive for HP infection, HP eradication should follow standard practice, repeating the UBT after one month to demonstrate that treatment was effective. Because HP infection is the major cause of gastric cancer, eradication of infection should be the most effective method to prevent its occurrence [47]. However, only few studies have reported the effects of screening and treating this pathogen at the population levels [66], given the lack of infrastructures for delivery of systematic screening services, the lack of standardization to ensure that each subject receives the correct diagnostic testing and antibiotic treatment, and limitation of resources.

If patients have low serum vitamin B_12_ and iron concentrations, their replacement should obviously be effected. In addition, in patients negative for pernicious anemia it is advisable to repeat the screening tests yearly, in that pernicious anemia or gastritis may appear later on.

During regular follow-up for CVID, patients with low serum levels of vitamin B_12_, patients with positive UBT and those with dyspeptic symptoms or unexplained weight loss should undergo upper gastrointestinal endoscopy, including biopsies of the antrum and fundus. Patients with premalignant lesions should receive endoscopic surveillance. 

In the absence of established guidelines, it seems reasonable to adopt the following procedure: (a) no follow-up endoscopy in patients with normal histopathology and (b) repeat endoscopy after a time interval ranging from a few months to 5 years, depending on the histopathological diagnosis [67,68] (Figure 2). The time intervals for follow-up of gastric precancerous lesions are based upon data on estimated rates of progression to gastric cancer. Progression rates to cancer vary from: 0 to 1.8% per year for atrophic gastritis, 0 to 10% per year for intestinal metaplasia, and 0 to 73% per year for dysplasia [69]. Obviously, the time intervals of follow-up should be personalized in each individual patient depending on location, severity and extent of gastric pathology and the occurrence of other risk factors for gastric cancer. 

## 6. CVID and Gastric Lymphoma

Lymphoproliferative disorders are common in CVID. Gastrointestinal lymphoid hyperplasia and/or splenomegaly are found in at least 20% of the patients with CVID [70]. Splenomegaly has in fact been reported in 26% of a cohort of 2212 European patients [15]. The cause of CVID-associated gastrointestinal lymphoproliferation is not known, but the potential role of bacterial, protozoal (mainly *Giardia lamblia*), and viral gastrointestinal infections should be kept in mind. Their eradication may be difficult for some patients.

Biopsies of lymph nodes usually show atypical or reactive lymphoid hyperplasia, but granulomatous inflammation may also be found. Typical features are the lack of plasma cells and the presence of ill-defined germinal centers in lymph nodes and other lymphoid tissues [71]. These same tissues should be examined for B- and T-cell clonality, using fluorescence markers, cytogenetics, and/or molecular analysis to rule out lymphoid malignancy. In lymph nodes with B-cell infiltrates, examination for EBV-encoded RNAs by in situ hybridization should be performed, often showing an expansion of transitional CD19^+^CD38^++^IgM^high^ B cells or CD19^+^CD38^low^CD21^low^ B cells [72]. Given that patients with CVID may have unusual lymphoid structures with loss of characteristic boundaries, it is important that the biopsies be examined by an experienced pathologist, in that the presence of clonal lymphocytes is not in itself diagnostic of lymphoma because these cells can be found in CVID lymphoid tissue showing reactive hyperplasia [70].

The risk for lymphoma in CVID is estimated to lie between 1.4% and 7% [16,44,45,73]. About 2–8% of subjects with CVID are diagnosed with NHL, in step with the longer survival of these patients [70]. In a study carried out on 248 consecutive CVID patients, who have been followed-up for 1–25 years, 23 patients were diagnosed with lymphoid malignancies. Specifically, 19 patients had NHL, three Hodgkin’s disease, and one Waldenström’s macroglobulinemia [74]. In this context, it is worth emphasizing that: (a) NHL occurs rarely in the pediatric population [75] and (b) in most cases it is usually B cell in type, extranodal, EBV-negative and more frequent in females than males [76]. A study of 98 CVID patients who have been followed-up for periods of 1–13 years showed an eight- to 13-fold increase in cancer in general and a 438-fold increase in lymphoma for females [76]. An earlier report based on a European cohort of 176 patients found three of the four NHL in women [45]. 

Extranodal marginal zone NHL arising in mucosal sites, named also mucosa-associated lymphoid tissue (MALT) lymphomas or “maltomas”, can also affect CVID patients. In the earlier literature, 10 cases of extranodal marginal zone lymphoma complicating CVID have been reported [73], but probably many more cases are clinically hidden. Extranodal marginal zone lymphomas are low-grade B cell lymphomas that occur in organs with lymphoid infiltration, due to long-term infectious or autoimmune stimulation [14]. A causal relationship is likely to exist between HP infection and extranodal marginal zone lymphoma with gastric location, in that HP infection is present in more than 90% of the patients with this type of lymphoma [77]. 

Finally, a subset of CVID patients with T cell lymphoma should be mentioned. Gottesman et al. described the occurrence of a peripheral extranodal T cell lymphoma arising in the bone marrow, liver and central nervous system of a patient with CVID. Immunohistochemical phenotyping and gene rearrangement studies revealed a T cell origin of this lymphoma. It was not associated with EBV infection of the lymphoma cells [78]. Recently, Jesus et al. illustrated a case of CVID associated with hepato-splenic T-cell lymphoma mimicking juvenile systemic lupus erythematosus. The autopsy showed a diffuse involvement of bone marrow, spleen, liver, and lungs. The lymphoma cells were positive for CD3 and negative for CD20 and lysozyme expression [79]. CD8^+^ granulomatous cutaneous T-cell lymphoma is a rarely encountered entity that appears to be associated with immunodeficiency, as reported by Gammon et al. in a retrospective review of four cases. Patients were characterized by an asymptomatic papulo-nodular eruption occurring in association with immunodeficiency [80]. 

Usually, patients with splenomegaly alone may not need treatment. Likewise, persistent hypertrophy of lymph nodes should suggest to review the diagnosis in order to exclude lymphoma but, again, this does not imply that treatment should be given. The administration of corticosteroids is commonly associated to regression of these phenomena, but they may recur when steroids are tapered. A rapid increase in adenopathy or splenomegaly should prompt evaluation for possible malignant transformation. Lymphoma may be difficult to distinguish from polyclonal lymphoid proliferation. Clonal analysis can be misleading because oligoclonal lymphocyte subpopulations have been found in biopsies, irrespective of histology [70]. Treatment follows the current protocols for immunocompetent patients.

## 7. Conclusions

CVID seems to be a predisposing factor to gastric malignancies. The reasons for this increased susceptibility are still unclear and additional animal models of CVID are needed to establish mechanisms of its relationship to cancer. The impaired immunity to potentially carcinogenic pathogens, the weakened tumor cell surveillance, and T and B cell defects are no doubt predisposing factors. 

Due to the great heterogeneity of CVID patients, there are no set rules regarding their therapy and follow-up. Although treatment obviously requires the infusion of human immunoglobulins, an unsettled point is whether an adequate immunoglobulin replacement is sufficient to prevent the increased risk of malignancy. Bacterial and viral infections must be treated. Low-dose corticosteroids can be administered to ameliorate gastrointestinal lymphoproliferative disorders, but higher doses should be avoided to prevent the risk of opportunistic infections [12]. Treatment of gastric cancer and lymphoma must follow the current protocols for immunocompetent patients. Further studies are recommended to better identify patients at high risk of gastric neoplasias and to better treat them.

## Figures and Tables

**Table 1:** Genetic immune defects in common variable immunodeficiency.

Gene	Defect	References
*TNFRSF13B*	Homozygous and heterozygous mutations	[18,19,24]
*BAFF-R*	Homozygous and heterozygous mutations	[25]
*CD20*	Homozygous mutations	[29]
*CD19-B-cell receptor complex*	Homozygous mutations	[30,31]
*ICOS*	Homozygous deletions	[32]
Genes implicated in DNA repair *(MSH5*, *MSH2*, *MLH1*, *RAD50* and *NBS1)*	Heterozygous non-synonymous mutations	[35]
*CARD11*	Heterozygous single nucleotide polymorphisms	[36]
*Bob1*	Heterozygous single nucleotide polymorphisms	[36]
*MHC region*	Single nucleotide polymorphisms	[37]
*ADAM*	Single nucleotide polymorphisms	[37]
*CTLA4*	Heterozygous nonsense mutations Frameshift deletion Intronic mutations	[38]
*PIK3CD*	Heterozygous splice site mutations Gain-of-function mutations	[39]
*NFκB2*	Heterozygous frameshift mutation Heterozygous nonsense mutation	[40]
*PLCG2*	Deletions	[41]
*LRBA*	Homozygous mutations	[42]
*CD27*	Homozygous mutations	[43]
